# LncRNA DGCR5 Isoform-1 Silencing Suppresses the Malignant Phenotype of Clear Cell Renal Cell Carcinoma via miR-211-5p/Snail Signal Axis

**DOI:** 10.3389/fcell.2021.700029

**Published:** 2021-07-12

**Authors:** Guang-Xin Zhong, Dan Luo, Yi-jun Fan, Jue Wang, Bing-Qiang Liu, Zhong-Hua Xu, Xiang Zhang

**Affiliations:** ^1^Department of Urology, Qilu Hospital of Shandong University, Jinan, China; ^2^School of Medicine, Shandong University, Jinan, China; ^3^Institute of Medical Science, Central Research Laboratory, The Second Hospital of Shandong University, Jinan, China; ^4^School of Mathematics, Shandong University, Jinan, China

**Keywords:** DGCR5, cell proliferation, ceRNA, clear cell renal cell carcinoma, miR-211-5p

## Abstract

Long non-coding RNAs (lncRNAs) play important roles during the initiation and progression of cancer. We identified DiGeorge Syndrome Critical Region Gene 5 (DGCR5) as a clear cell renal cell carcinoma (ccRCC) cancer- and lineage-specific lncRNA. Agarose gel electrophoresis analysis and sanger sequencing verified two main isoforms of DGCR5 in ccRCC patient tissues and cell lines. Quantitative polymerase chain reaction further demonstrated that the expression level of DGCR5 major isoform (isoform-1) was higher in ccRCC tissues than that in papillary/chromophobe RCC and other multiple solid malignant tumors. We investigate the biological functions of DGCR5 isoform-1 in ccRCC and show that DGCR5 isoform-1 exerts a tumor-promoting effect in ccRCC. DGCR5 isoform-1 is localized in cytoplasm and shares the same binding sequence to the tumor-suppressive miR-211-5p with the epithelial-to-mesenchymal transition key component SNAI. Furthermore, cellular and molecular experiments demonstrate that DGCR5 isoform-1 could sequester miR-211-5p, leading to the elevation of Snail protein and downregulation of its downstream targets and further promoting ccRCC cell proliferation and migration. Thus, our study indicates that DGCR5 isoform-1 could contribute to ccRCC progression by sponging miR-211-5p through regulating the expression of Snail protein and could serve as a reliable diagnostic biomarker in ccRCC.

## Introduction

Clear cell renal cell carcinoma (ccRCC) is the most common kidney cancer in adults and also the most fatal cancer among all urinary malignant tumors (Bray et al., 2018; Siegel et al., 2018). Patients with ccRCC are usually asymptomatic, and due to its asymptomatic nature, a considerable number of patients are initially diagnosed with regional advanced disease or even with distant metastatic lesions. The 5-year survival rate of ccRCC patients with distant metastatic disease is less than 12% according to the Surveillance, Epidemiology, and End Results Program. Early diagnosis and treatment are of vital importance to improve the outcome of ccRCC. Owing to the low incidence of RCC, developing accurate biomarkers addresses an unmet need.

Long non-coding RNAs (lncRNAs) have more than 200 bases in length and limited protein-coding capability. They actively participate in various cellular activities, including carcinogenesis. Some LncRNAs present in certain cancers with lineage-specific expression patterns, which make lncRNAs useful as cancer biomarkers (Iyer et al., 2015). Our previous data mining and analysis of publicly available RNA sequencing data identified DiGeorge Syndrome Critical Region Gene 5 (DGCR5) as a ccRCC cancer- and lineage-specific expressed lncRNA, which highly expresses in ccRCC human tissues (Zhang et al., 2018). Recent studies show that DGCR5 plays important roles in various types of cancers in the lung (Chen et al., 2017; Dong et al., 2018; Luo et al., 2018; Wang R. et al., 2018), liver (Wang Y.G. et al., 2018; Wang et al., 2019), stomach (Xu et al., 2019), and bladder (Fang et al., 2019), among others. Both tumor-promoting and -suppressing effects of DGCR5 are reported in different types of cancers (Huang et al., 2016; Chen et al., 2017; Dong et al., 2018; Luo et al., 2018; Wang R. et al., 2018; Wang Y.G. et al., 2018; Fang et al., 2019; Liu et al., 2019; Tang and Shan, 2019; Tang et al., 2019; Xu et al., 2019) or even in the identical types of cancer (Chen et al., 2017; Dong et al., 2018; Luo et al., 2018; Wang R. et al., 2018). However, the biological role of DGCR5 in ccRCC, although it is most highly expressed in ccRCC among the abovementioned cancer types, remains unclear.

This study investigates the isoforms of DGCR5 and demonstrates that the silencing of DGCR5 isoform-1 suppresses the malignant phenotype of ccRCC. DGCR5 isoform-1 could sponge the tumor-suppressive miR-211-5p to regulate the expression of Snail protein as well as its downstream targets. Therefore, our study first identified the isoform-1 of DGCR5 in ccRCC, which has the potential to be a reliable diagnostic biomarker as well as a novel therapeutic target in ccRCC management.

## Materials and Methods

### Statement of Ethics

This study was approved by the Ethics Committee of Qilu Hospital of Shandong University. Signed patient consents were obtained from all participants in this study.

### Patient Specimens

Sixteen pairs of ccRCC cancer tissues and adjacent non-cancerous tissues collected from ccRCC patients during surgery at the Qilu Hospital of Shandong University between May 2018 and September 2018 were immediately snap-frozen and stored in liquid nitrogen until use. No patients had received radiotherapies or chemotherapies prior to surgery.

### Cell Culture

The human RCC cell lines (A498, A704, 786-O, and Caki-1) and immortalized normal human proximal tubule epithelial cell line HK-2 were purchased from American Type Culture Collection (Manassas, VA, United States). A498 and A704, 786-O and HK-2, Caki-1 and HEK-293 were cultured, respectively, in modified Eagle’s medium (MEM, Gibco, Thermo Fisher Scientific, Waltham, MA, United States), RPMI-1640 medium (Gibco), McCoy’s 5A medium (Gibco), and Dulbecco’s MEM (Gibco) supplemented with 10% fetal bovine serum (FBS, Biological Industries, Beit HaEmek, Israel) at 37°C in a humidified incubator containing 5% CO_2_.

### RNA Extraction, Reverse-Transcription PCR, and Real-Time Quantitative PCR

Total RNA was extracted from cells or tissues with TRIzol (Invitrogen, Carlsbad, CA, United States) according to the manufacturer’s protocols. The concentration and purity of RNA were determined by NanoDrop ND2000 (Thermo Fisher Scientific Inc., Santa Clara, CA, United States). The reverse transcription of RNA was performed using a ReverTra Ace qPCR RT Master Mix with gDNA Remover (Toyobo, Osaka, Japan). The quantitative PCR was carried out with SYBR Green Realtime PCR Master Mix (Toyobo) on a CFX96 Real-Time System thermal cycler (Bio-Rad, Hercules, CA, United States) in accordance with the manufacturer’s instructions. The primers used were synthesized by GenePharma Co., Ltd. (Shanghai, China) and are listed in Supplementary Table 1. All mRNA expression levels were normalized to GAPDH, and relative expression was measured by the 2^–Δ^ ^Δ^ ^*CT*^ method. All reactions were tested in triplicate.

### Agarose Gel Electrophoresis

The agarose gel was made by the heating of agarose powder (BaygeneBio, Shanghai, China) and Tris-acetate-EDTA (TAE, Solarbio, Beijing, China) buffer followed by mixing with GelRed (Mei5Bio, Beijing, China). Electrophoresis was performed in 1% TAE running buffer at 90 V for 50 min. The results were obtained and analyzed by an ultraviolet transilluminator with Image Lab software (Bio-Rad, Hercules, CA, United States).

### Subcellular Fractionation Followed by Quantitative PCR

Nuclear/cytoplasmic subcellular fractionation of DGCR5 in A704 cells was performed using the NE-PER Nuclear and Cytoplasmic Extraction Kit (Thermo Fisher Scientific, Waltham, MA, United States) according to the manufacturer’s instructions. qRT-PCR was carried out to assess the expression of DGCR5 in nuclear and cytoplasm. The cytoplasmic and nuclear expression of DGCR5 was normalized to β-actin and U1, respectively.

### Cell Transfection

SiRNAs targeting DGCR5 (siDGCR5), FAM-siRNA, and negative-control siRNA (siNC) as well as miRNA negative controls (miR-control) and miR-211-5p mimics and inhibitors were designed and synthesized by GenePharma Co., Ltd. (Shanghai, China), and the sequences are listed in Supplementary Table 2. A704 cells were transfected with RNA products using Lipofectamine 2000 transfection reagent (Invitrogen, Carlsbad, CA, United States) per the manufacturer’s instruction. RNA or protein was isolated after 48 and 72 h after transfection.

### Cell Proliferation Assay

Post-transfected A704 cells were seeded 5000 cells per well in 96-well plates. The cell counting kit-8 (CCK-8, Dojindo, Kumamoto, Japan) assay was performed to analyze the cell viability at time points 24, 48, 72, and 96 h. Optical density was measured at 450 nm using a microplate reader (SpectraMax; Molecular Devices, San Jose, CA, United States).

### EdU Assay

The transfected A704 cells were seeded 3 × 10^4^ cells per well in 24-well plates and cultured for 24 h. The proliferation of A704 cells was detected using a 5′-ethynyl-2′-deoxyuridine (EdU) kit (RiboBio, Guangzhou, China) per the manufacturer’s instructions. The percentage of positive cells stained with both EdU and Hoechst was used to compare cell proliferation capabilities in different groups.

### Cell Cycle Analysis

A704 cells post-transfection for 48 h were washed with cold PBS three times, stained with the BBcellProbe Kit (BestBio, Shanghai, China) according to the manufacturer’s protocols, and fixed with 75% ethanol overnight at 4°C. The cell cycle was analyzed by flow cytometer (BD Biosciences, United States). The experiments were performed in triplicate.

### Transwell Invasion Assay

Transfected A704 cells were harvested, suspended with serum-free MEM medium, and seeded at a density of 4 × 10^4^ cells per well into the Transwell upper chambers (8 μm; Millipore, Billerica, MA, United States) coated with Matrigel (BD, Bedford, MA, United States). Then, 700 μl complete media was added to the lower chambers. After incubation for 48 h at 37°C in a 5% CO_2_ incubator, cells were fixed by methanol and stained with 0.1% crystal violet. The invasive cells were counted in five random fields (×100) for quantification after wiping off non-invasive cells of the upper chambers. The assays were performed in triplicate.

### Transwell Migration Assay

A704 cells were harvested, suspended with serum-free MEM medium, and seeded with a density of 3 × 10^4^ cells per well into the Transwell upper chambers (8 μm; Millipore). Medium supplemented with 10% FBS was placed in the lower chamber. The non-migrated cells were carefully wiped out after incubation for 24 h. The migrated cells were fixed with methanol, stained with 0.1% crystal violet, and counted in five random fields (×100). The tests were performed in triplicate.

### Wound Healing Assay

A704 cells were seeded into six-well plates after 48 h of transfection, and the monolayer was carefully scratched with sterile 200 μL pipette tips after reaching 90–95% confluence. After removing floating cells with PBS, wound closure was obtained at 0 and 24 h. The percentage of wound closure calculated by the healing wound width at 24 h to the initial wound width at 0 h was used to analyze the cell migration abilities in various groups. Experiments were performed in triplicate.

### Bioinformatics Analysis

The potential miRNA targets of DGCR5 were identified using the miRcode database (Jeggari et al., 2012) and DIANA-LncBase v2 (Paraskevopoulou et al., 2016). The common putative targets in both databases were selected and depicted as a Venn diagram by Venn web tools^1^. The Oncomine database with Jones (Jones et al., 2005) and Yusenko (Jones et al., 2005) data was used to analyze the expression of DGCR5 in RCC and normal kidney tissues^2^.

### Luciferase Reporter Assay

The mutant (MUT) or wild-type (WT) miRNA response elements (MREs) for miR-211-5p of DGCR5 sequences were synthesized and cloned into a pGL3 basic vector (Promega, Madison, WI, United States). The reporter plasmids and miR-211-5p mimics were cotransfected into HEK-293 cells using Lipofectamine 2000 transfecting reagent (Invitrogen, Carlsbad, CA, United States). The luciferase activity was examined after transfection of 48 h by the Dual-Luciferase Reporter Assay System (Promega) according to the manufacturer’s instructions. Renilla luciferase activity was used for normalization.

### Western Blot Analysis

Total protein was extracted from transfected cells using RIPA buffer (Beyotime, Haimen, China) containing PMSF (Beyotime) according to the manufacturer’s protocols. After determination of protein concentration by BCA kits (Beyotime), 20-μg protein samples were separated by sodium dodecyl sulfate polyacrylamide gel electrophoresis and transferred onto polyvinylidene difluoride membranes (Millipore, Billerica, MA, United States) followed by blocking in Tris-Buffered Saline Tween-20 with 5% bovine serum albumin (Beyotime). The membranes were incubated at 4°C overnight with specific primary antibodies: Snail (1:1000, Cell Signaling Technology), β-actin (1:1000, Abcam, Cambridge, MA, United States). After incubating with HRP-conjugated secondary antibody (1:2500, Zsgb-Bio, Beijing, China), protein bands were visualized using Chemiluminescent HRP Substrate (Millipore) on a Western blot imaging system (Amersham Imager 600, GE Amersham, United States). β-actin was used for normalization.

### Statistical Analysis

All quantitative data were presented as mean ± SD. All statistical analyses were carried out with SPSS version 17.0 (Abbott Laboratories, Chicago, IL, United States). Student’s *t*-test was used for comparison of two groups, and one-way analysis of variance (ANOVA) followed by Tukey’s *post hoc* test was performed for multiple comparisons. *P* < 0.05 was considered as statistically significant.

## Results

### LncRNA DGCR5 Expression Is Significantly Upregulated in Human ccRCC Cell Lines and Tissues

**FIGURE 1:**
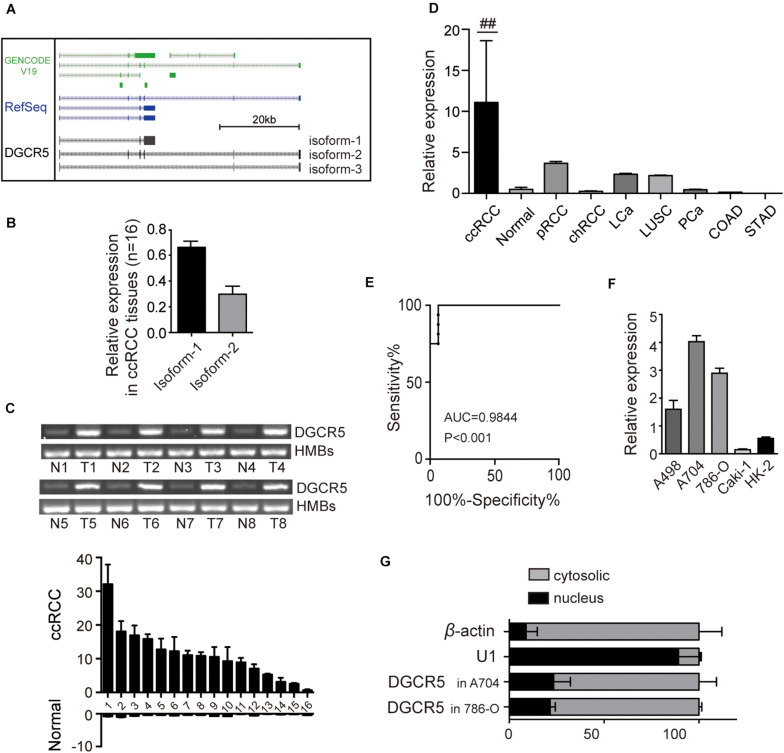
The expression of DGCR5 isoforms in RCC cell lines. **(A)** The cartoon graph displays the main isoforms of DGCR5 predicted by the UCSC gene browser and validated by this study using spanning PCR with Sanger sequencing. **(B)** The two main isoforms of DGCR5 were tested using agarose gel electrophoresis, and their expression levels are shown. **(C)** The expression of DGCR5 in freshly frozen ccRCC and the matched normal tissues was tested by agarose gel electrophoresis. HMB was used as internal control. **(D)** The relative DGCR5 expression was detected by qPCR in ccRCC, normal renal tissues, pRCC, and chRCC tissues as well as LCa, LUSC, PCa, COAD, and STAD tissues. GADPH was used as internal controls. **(E)** The ROC curve based on DGCR5 expression in ccRCC patients. **(F)** The expression of DGCR5 in RCC cell lines A498, A794, 786-O, and Caki-1 and the immortalized renal epithelial cell line HK-2. qPCR was performed to test the DGCR5 expression in RCC cell lines. **(G)** Subcellular localization of DGCR5 in A704 and 786-O cells, tested by subcellular fractionation followed by qPCR. Three independent experiments were carried out. Data are shown as mean ± SD; ^##^*P* < 0.0001. DGCR5, DiGeorge syndrome critical region gene 5; qPCR, quantitative polymerase chain reaction; RCC: renal cell carcinoma; ccRCC, clear cell renal cell carcinoma; N, normal; T, tumor; pRCC, papillary renal cell carcinoma; chRCC, chromophobe renal cell carcinoma; LCa, liver cancer; LUSC, lung squamous cell carcinoma; PCa, prostate cancer; COAD, colon adenocarcinoma; STAD, stomach adenocarcinoma; ROC, receiver operator characteristic; AUC, the Area Under Curve; SD, standard deviation.

To determine the expression of lncRNA DGCR5 in human RCC cell lines and tissues, we first screen for PCR primers targeting different exons of DGCR5 (primer sets are illustrated in Supplementary Figure 1A and Supplementary Table 1) and found the expression levels of DGCR5 in different cell lines were varied using different primer sets (Supplementary Figure 1B), indicating several isoforms may exist. Next, we explored the full-length sequence of DGCR5 by performing spanning PCR and Sanger sequencing. The results show that, different from the UCSC gene browser prediction, there are two main isoforms of DGCR5, with isoform-1 (major isoform) accounting for 65% of its abundance, and isoform-2 accounting for 30% (Figures 1A,B). Our preliminary experiments show it was difficult to design siRNAs that could effectively knock down isoform-2 (the one with six exons as shown in Figure 1A). In subsequent experiments, we focused on isoform-1 using primer set 3, which exclusively targets DGCR5 isoform-1 (The full-length sequence of isoform-1 is provided in Supplementary Figure 1C). The DGCR5 expression was detected in freshly frozen ccRCC, papillary RCC, and chromophobe RCC human tissues and RCC cell lines using qPCR. DGCR5 expression was significantly upregulated in ccRCC compared with normal renal tissues (*P* < 0.0001, Figures 1C,D). However, DGCR5 expression was very low in papillary RCC, liver cancer, and lung squamous cell carcinoma and rarely detected in chromophobe RCC, prostate cancer, and colon cancer (Figure 1D). The area under the curve of DGCR5 expression was 0.9844 in ccRCC patient tissues (Figure 1E). Additionally, DGCR5 expression was also significantly upregulated in human RCC cell lines A704, 786-O, and A498 compared with the immortalized renal epithelial cell line HK-2 with the highest expression of DGCR5 in A704 (Figure 1F). Subcellular fractionation followed by qPCR analysis demonstrated that DGCR5 was mainly localized in the cytoplasm of A704 and 786-O cells (Figure 1G).

### DGCR5 Isoform-1 Silencing Suppressed ccRCC Cell Proliferation, Migration, and Invasion and Caused Cell Cycle Arrest in S Phase

**FIGURE 2:**
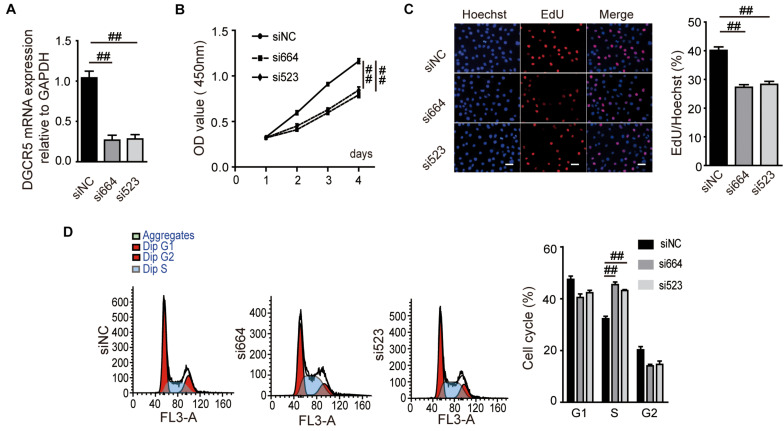
Effects of DGCR5 isoform-1 on RCC cell proliferation and cell cycle. **(A)** DGCR5 mRNA expression detected by qPCR in A704 cells. Cells were transfected with si664- or si523-targeting DGCR5 or siRNA-NC for 48 h. The mRNA expression of DGCR5 was normalized to GAPDH. Effects of DGCR5 knockdown on cell viability, cell proliferation, and cell-cycle regulation, which is detected by CCK8 assay **(B)**, EdU assay (**C**, magnification ×100), and cell flow cytometry analysis **(D)**, respectively. All experiments were performed in triplicate and repeated in A704 cells. Data are shown as mean ± SD; ^##^*P* < 0.0001. siNC, small interfering RNA negative control; si664, small interfering RNA 664 targeting DGCR5; si523, small interfering RNA 523 targeting DGCR5; CCK8, cell counting kit-8; OD, optical density; EdU, 5-ethynyl-2′-deoxyuridine.

**FIGURE 3:**
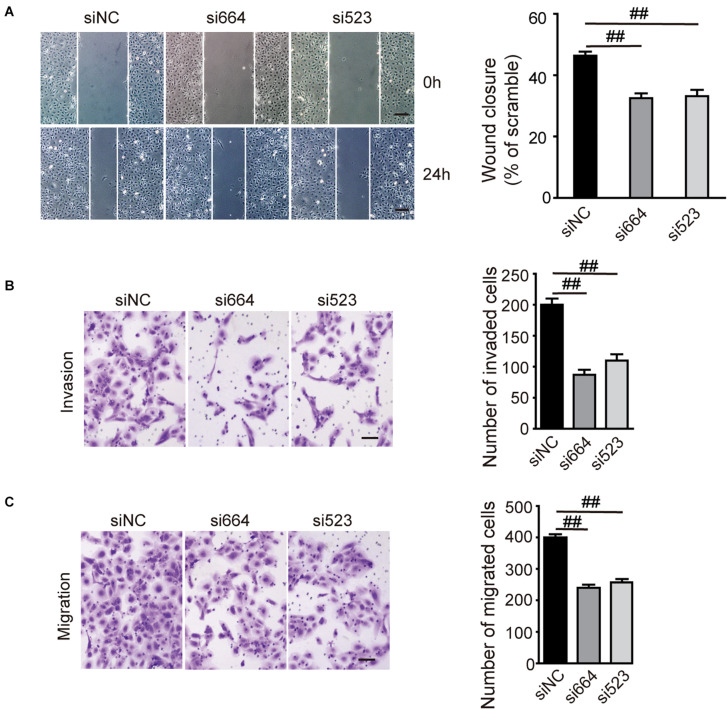
Effects of DGCR5 isoform-1 on RCC cell invasion and migration. **(A)** Effects of DGCR5 knockdown on cell invasion and migration, which is detected by matrigel cell invasion assay (**A**, magnification ×100), transwell cell migration assay **(B)**, and wound healing assay (**C**, magnification ×100), respectively. Experiments were performed in triplicate. Data are shown as mean ± SD;^ ##^*P* < 0.0001.

To investigate the biological function of DGCR5 in ccRCC cell lines, we conducted loss-of-function experiments of DCGR5 and found that transfecting si664 and si523 in A704 cells significantly downregulated DCGR5 isoform-1 expression (Figure 2A and Supplementary Figures 2A,B) and remarkably suppressed cell proliferation (Figure 2B). Consistently, the percentage of EdU-positive cells was significantly decreased in si664- or si523-treated A704 cells compared with normal controls (Figure 2C). Moreover, flow cytometry analysis revealed a significant increase in S phase of the si664- or si523-transfected A704 cells than the siNC-transfected groups (Figure 2D). Furthermore, matrigel cell invasion and transwell cell migration assays showed that fewer cells penetrated through the chamber membrane in A704 cells transfected with si664 and si523 compared with the control group (Figures 3A,B). Congruously, the wound healing assay also showed that a smaller percentage of wound closure was observed in A704 cells transfected with si664 and si523 compared with controls (Figure 3C). Similar findings were also observed in the si664-transfected 786-O and A498 cells compared with controls (Supplementary Figures 3A–D). These data demonstrate that DGCR5 silencing could significantly inhibit cell growth, invasion, and migration of human RCC cells *in vitro*.

### MiR-211-5p Served as a Direct Target of DGCR5 Isoform-1

**FIGURE 4:**
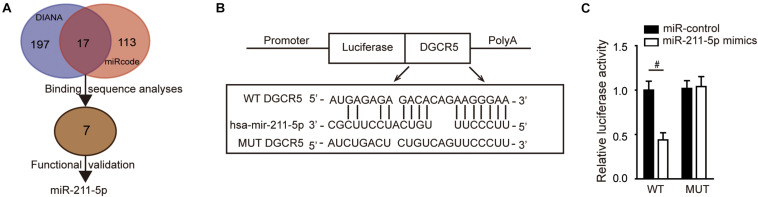
DGCR5 isoform-1 targets miR-211-5p *in vitro*. **(A)** The flow diagram of screening and validation of potential miRNA targets of DGCR5. **(B)** The luciferase reporter plasmids were constructed containing WT or MUT MREs for miR-211-5p of the DGCR5 sequence. **(C)** The luciferase reporter assay was analyzed in HEK293 cells cotransfected with plasmids containing WT or MUT MREs of DGCR5 sequence and miR-211-5p mimics. Independent experiments were carried out in triplicate. Data are shown as mean ± SD; ^#^*P* < 0.001. miR, microRNA; WT, wild type.

To study the molecular mechanisms of DGCR5-mediated tumor-promoting effects in human RCC, we analyzed potential miRNA targets of DGCR5 using the miRcode database (Supplementary Table 3) and DIANA-LncBase v2 (Supplementary Table 4). Seven miRNAs among 17 identified overlap miRNAs (Supplementary Table 5) were selected for further validation after the binding sequence analyses (Figure 4A). Next, cell proliferation assays for functional validation were used to screen for potential ccRCC-suppressing miRNAs, and it turned out that miR-211-5p is the best candidate gene (data not shown). To validate the direct binding, a luciferase reporter gene assay was performed, and the constructed luciferase reporter plasmids containing MREs for miR-211-5p of the DGCR5 sequence are shown in Figure 4B. The luciferase activity was significantly decreased after being cotransfected with miR-211-5p mimics in the WT MRE of the DGCR5 group but not the MUT group (*P* = 0.0006; Figure 4C), which confirmed the direct binding between DGCR5 and miR-211-5p.

### MiR-211-5p Inhibition Increased ccRCC Cell Proliferation and Migration

**FIGURE 5:**
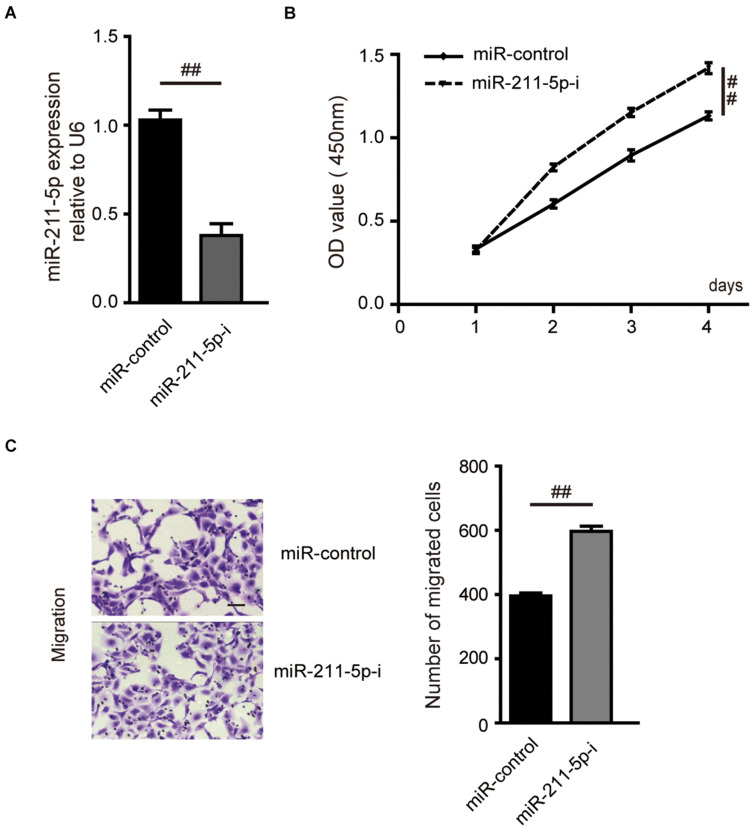
Effects of miR-211-5p on RCC cell proliferation and migration. **(A)** The mRNA expression of miR-211-5p was assayed by qPCR in A704 cells. Cells were transfected with miR-control or miR-211-5p inhibitors for 48 h. U6 was used as normalization. Effects of miR-211-5p on the cell viability and migration, which is detected by CCK8 assay **(B)** and transwell migration assay **(C)**, respectively. Independent experiments were carried out in triplicate in A704 cells. Data are shown as the mean ± SD; ^##^*P* < 0.0001.

MiR-211-5p expression was significantly downregulated in A704 cells transfected with miR-211-5p inhibitors compared with the control group (Figure 5A). miR-211-5p inhibition significantly suppressed the A704 cell proliferation measured by CCK8 assay (Figure 5B). In addition, a transwell cell migration assay showed that more cells penetrated through the chamber membrane in A704 cells transfected with miR-211-5p inhibitors compared with the control group (Figure 5C).

### The Tumor-Suppressing Effect Through DGCR5 Isoform-1 Silencing Was Reversed by miR-211-5p Inhibition

**FIGURE 6:**
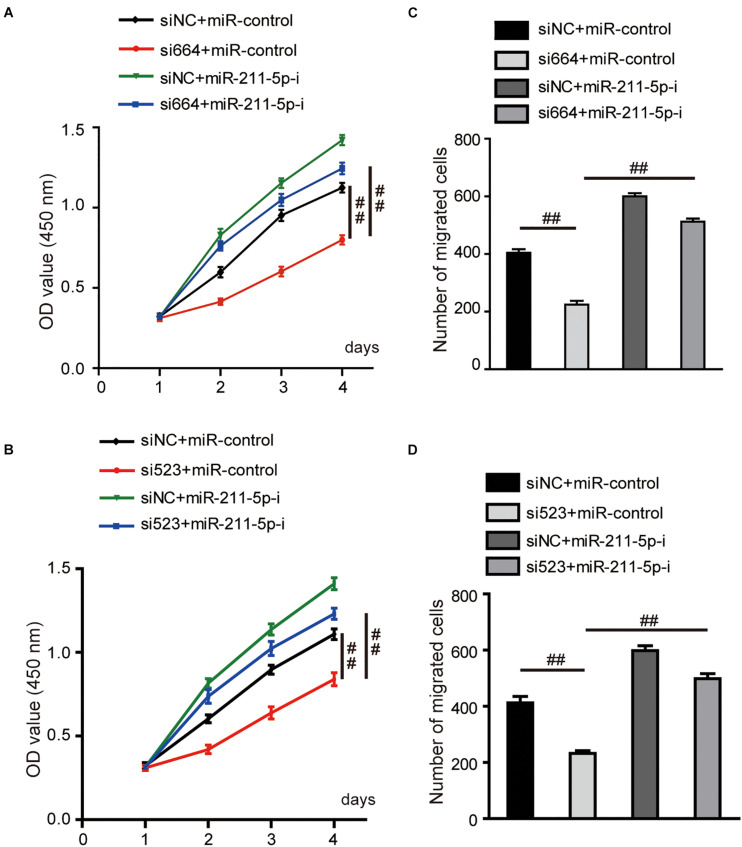
Effects of DGCR5 isoform-1 on RCC cell viability and migration by targeting miR-211-5p. **(A,B)** The CCK8 assay was carried out to detect the cell viability of A704 cells transfected with siRNA targeting DGCR5 (si664 or si523), miR-211-5p inhibitors or both siRNA and miR-211-5p inhibitors. **(C,D)** The transwell cell migration assay was carried out to test the migratory capability of A704 cells transfected with siRNA targeting DGCR5 (si664 or si523), miR-211-5p inhibitors or both siRNA and miR-211-5p inhibitors. All experiments were repeated in triplicate. Error bars stand for the mean ± SD; ^##^*P* < 0.0001. miR-211-5p-i, microRNA-211-5p inhibitors.

The ability of cell proliferation was significantly decreased by DGCR5 silencing in A704 cells transfected with si664 or si523, and this inhibitory effect was reversed by transfection of miR-211-5p inhibitors (Figures 6A,B). Consistently, the number of migrated cells was remarkably decreased in A704 cells treated with si664 or si523 although, again, this inhibitory effect was reversed by transfection of miR-211-5p inhibition (Figures 6C,D).

### DGCR5 Isoform-1 Increased Snail Expression by Sequestering miR-211-5p

**FIGURE 7:**
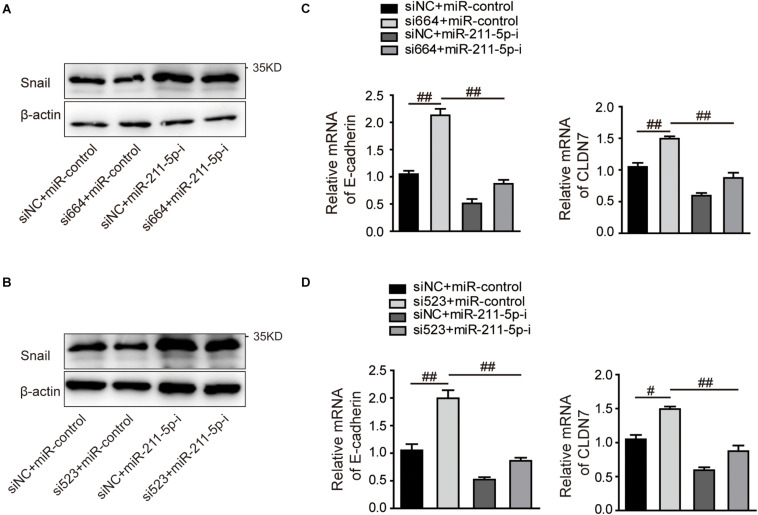
The effects of DGCR5 isoform-1 on RCC cell Snail expression and its downstream gene transcription by targeting miR-211-5p. Western blot was performed to detect Snail protein expression **(A,B)**, and qPCR was performed to detect E-cadherin and CLDN7 mRNA expression **(C,D)** in A704 cells treated with siRNA targeting DGCR5 (si664 or si523), miR-211-5p inhibitors or both siRNA and miR-211-5p inhibitors. All experiments were performed in triplicate. Data are shown as mean ± SD; ^#^*P* < 0.001 and ^##^*P* < 0.0001.

**FIGURE 8:**
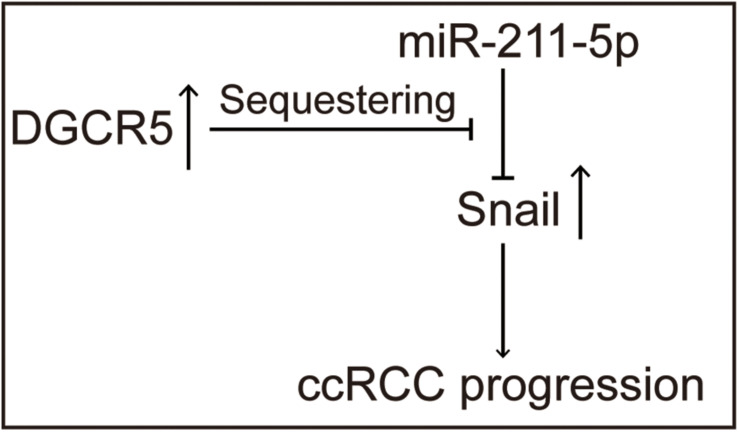
Schematic diagram shows the tumor-promoting roles of the DGCR5 isoform-1 in ccRCC progression via miR-211-5p/Snail signal axis.

Previous studies demonstrate that miR-211-5p could downregulate Snail protein and suppress RCC cell migration. We examined the effect of DGCR5 isoform-1 on Snail protein expression by Western blot analysis. The results show that the Snail protein level was significantly decreased in the A704 cells transfected with DGCR5 si664 or si523 inhibitors and increased in the A704 cells transfected with miR-211-5p inhibitor (Figure 7A). In knockdown A704 cells by DGCR5 si664 or si523 inhibitors, the diminished Snail protein level was reversed by transfecting miR-211-5p inhibition (Figure 7B). Furthermore, qPCR showed that DGCR5 isoform-1 knockdown by si664 or si523 in the A704 cells could upregulate Snail downstream targets, E-cadherin, and CLDN (Figure 7C). This effect could be reversed by transfecting miR-211-5p inhibitors (Figure 7D). These results demonstrate that DGCR5 isoform-1 can upregulate Snail expression and promote the malignant phenotype by sponging miR-211-5p in ccRCC cells (Figure 8).

## Discussion

The prognosis of patients with ccRCC is favorable with a 5-year survival rate of more than 90% if there is no metastasis. However, the 5-year survival rate is drastically decreased to less than 12% after metastasis (Bray et al., 2018). Due to its asymptomatic nature, people with ccRCC are often diagnosed with advanced disease, and consequently, have a poor prognosis (Bray et al., 2018). In light of this, early diagnosis remains crucial in managing ccRCC, which highlights the necessity for the development of novel and reliable tumor biomarkers within the field of kidney cancer screening. Recent evidence supports the key role of lncRNAs in a variety of biological and pathological processes, including carcinogenesis (Prensner and Chinnaiyan, 2011). Moreover, due to the cancer- and lineage-specific expression pattern of lncRNAs, a group of lncRNAs have proven to be prospective biomarkers, e.g., PCA3 and PCAT14 in prostate cancer (Chun et al., 2009; Shukla et al., 2016), which could be used for the early detection, diagnosis, and follow-up in the clinic. The study conducted aims to screen for potential tumor biomarkers and therapeutic targets for ccRCC from a lncRNA perspective. We searched the publicly available RNA sequencing data and discovered that DGCR5 is a ccRCC-specific lncRNA.

Compared with protein-coding genes, lncRNA expression is generally lower, but their expression patterns are more tissue-specific (Iyer et al., 2015). A certain number of lncRNAs exhibit excellent cancer- and lineage-specific expression patterns; however, most of them only possess a pretty low expression level, which greatly raises the difficulty of lncRNA testing and hinders its use as a biomarker. DGCR5 is highly selectively expressed in ccRCC tissues, and at the same time, its expression level in ccRCC is quite high, which is one of the main reasons that we decided to further investigate its clinical use as a tumor biomarker. Recent studies demonstrate that DGCR5 is significantly dysregulated in gastric cancer (Xu et al., 2019), liver cancer (Wang Y.G. et al., 2018; Wang et al., 2019), and lung cancer (Chen et al., 2017; Dong et al., 2018; Luo et al., 2018; Wang R. et al., 2018), among others. However, our results show that the expression of DGCR5 is remarkably higher in ccRCC than in gastric, lung, liver, colon, and prostate cancer. Hence, ccRCC may represent an ideal model to observe the function of DGCR5.

Our study finds that DGCR5 is exclusively highly expressed in ccRCC although it is lowly expressed in papillary RCC (pRCC) and barely detected in chromophobe RCC (chRCC). Our results are consistent with the previous microarray studies (Jones et al., 2005; Yusenko et al., 2009). It is known that a certain fraction of pRCC exhibits indolent clinical behavior and a favorable prognosis. If the preoperative diagnosis could be achieved by tissue- or liquid-based biopsy, a more conservative treatment approach could be considered (Haddad et al., 2018). Recently, the molecular characterization of RCC by The Cancer Genome Atlas raised a challenge against the pathologic subtyping of ccRCC and pRCC (Chen et al., 2016). The differential diagnosis between ccRCC and pRCC, which is based on morphological features, and the immunohistochemical and molecular profiles, may sometimes be difficult. And DGCR5 examination may be of help in the differential diagnosis between ccRCC and pRCC. One limitation of our present study is that the sample size of pRCC is too small to effectively evaluate the differential diagnostic efficacy of DGCR5.

Similar to protein-coding genes, lncRNAs undergo active splicing, and various isoforms of lncRNAs exist (Iyer et al., 2015). When we designed primer sets to investigate the expression of DGCR5 in human ccRCC tissue samples, we got inconsistent results using different primer sets, suggesting that various DGCR5 isoforms may exist. We then performed spanning PCR using different primer sets, which is followed by gel retraction and Sanger sequencing to identify the potential DGCR5 isoforms in both cell lines and tissue samples. We found that, unlike the University of California, Santa Cruz (UCSC) genome browser prediction, there were two main DGCR5 isoforms in human ccRCC samples with the abundance of 65% and 30% for isoform-1 and isoform-2, respectively. Both main isoforms were ccRCC-specifically expressed. It is reported that DGCR5 expression in lung cancer patients and their sera are either upregulated (Dong et al., 2018; Wang R. et al., 2018) or downregulated (Chen et al., 2017; Luo et al., 2018). Those contradictory results could be due to the isoform-specific primers to distinguish DGCR5 isoforms not being used in the studies. We used isoform-specific primers and demonstrated that the major isoform (isoform-1) was specifically expressed in ccRCC with a high diagnostic value. We did not find, however, any associations of the DGCR5 isoform-1 expression level with any clinical parameters, such as TNM stage and Fuhrman nuclear grade, in patients with ccRCC (data not shown). In view of the small sample size of ccRCC specimens, isoform-specific test of DGCR5 in larger ccRCC cohorts with clinical information is warranted to evaluate its value as a prognostic biomarker.

The tumor-promoting or -suppressive role of DGCR5 during carcinogenesis is not clearly understood. DGCR5 is found to promote cell proliferation and cancer stem cell properties and contribute to the radioresistance in laryngeal carcinoma cells by sponging miR-506 and miR-195 (Tang and Shan, 2019; Tang et al., 2019). DGCR5 was upregulated in lung adenocarcinoma tissues and promoted cancer progression through inhibiting miR-22-3p (Dong et al., 2018), and the same team concluded that DGCR5 contributed to the cancer stem cell–like properties in non-small cell lung cancer through interacting with miR-330-5p/CD44 (Wang R. et al., 2018). However, other studies demonstrate a tumor-suppressive role of DGCR5 in lung cancer (Chen et al., 2017; Luo et al., 2018), gastric cancer (Xu et al., 2019), bladder cancer (Fang et al., 2019), and cervical cancer (Liu et al., 2019). It is reported that DGCR5 suppresses lung cancer cell proliferation, migration, and invasion by targeting miR-1180 (Chen et al., 2017) and by interacting with miR-873-5p to regulate the tumor suppressor candidate 3 (TUSC3) (Luo et al., 2018). Moreover, DGCR5 is found to function as an endogenous competing RNA for miR-23b to suppress gastric cell proliferation and invasion (Xu et al., 2019). In addition to this, DGCR5 downregulation is found to significantly correlate with the poor prognosis of lung cancer patients (Chen et al., 2017; Luo et al., 2018) and the aggressive clinical features in gastric cancer patients (Xu et al., 2019). Nevertheless, one common finding indicated in each of these studies is that DGCR5 is mainly localized in the cytoplasm and functions by direct binding to certain molecules.

We investigated the biological function of DGCR5 by conducting loss-of-function experiments *in vitro*. For unknown reasons, the knockdown of the second-major isoform (isoform-2) using an RNA interference strategy proved challenging. Therefore, we focused solely on the major isoform (isoform-1) of DGCR5. Consistent with the above findings, we also found that isoform-1 of DGCR5 was mainly localized in the cytoplasm in human ccRCC cells. Recently, multiple studies demonstrate that lncRNAs could modulate miRNA activities to further manipulate the expression of other RNAs *in trans* trough competitive endogenous RNA (ceRNA) interactions, which mainly occurs in the cytoplasm (Cesana et al., 2011; Tay et al., 2014; Thomson and Dinger, 2016). Thus, the primary focus was the ceRNA mechanism to unravel the underlying molecular mechanism.

We screened the potential targets for DGCR5 isoform-1 using bioinformatic tools (Jeggari et al., 2012; Paraskevopoulou et al., 2016), and we identified and validated that miR-211-5p was the DGCR5 isoform-1 direct target. Some previous studies show that miR-211-5p acts as a tumor suppressor in various cancers, including RCC (Wang et al., 2017; Quan et al., 2018), thyroid cancer (Wang L. et al., 2018; Liang et al., 2019), and liver cancer (Jiang et al., 2017). miR-211-5p is found to downregulate in metastatic RCC specimens, and miR-211-5p overexpression significantly inhibited cell migration and invasion via Snail protein downregulation *in vitro* (Wang et al., 2017). Snail plays a key role in the epithelial-to-mesenchymal transition (EMT) by acting as a transcriptional repressor (Santamaria et al., 2017). The Snail-induced EMT process involves the loss of E-cadherin (Cano et al., 2000), which is found to promote the metastatic phenotype of ccRCC (Mikami et al., 2011; Wang et al., 2017). Interestingly, our study shows that DGCR5 shares the same binding sequence with SNAI1 on miR-211-5p, and the downregulation of Snail protein by knocking down DGCR5 isoform-1 was reversed by miR-211-5p inhibition. Moreover, E-cadherin and CLDN7, the downstream targets of the transcriptional repressor Snail, was upregulated by knocking down DGCR5 isoform-1, and this upregulation was reversed by miR-211-5p inhibitors. Our findings provide evidence that DGCR5 isoform-1 could sponge the tumor-suppressive miR-211-5p to promote cell migration and invasion through regulating Snail in ccRCC cells *in vitro*.

## Conclusion

In conclusion, our study identified two main isoforms of DGCR5 in ccRCC and provided sufficient evidence that the DGCR5 major isoform (isoform-1) is exclusively expressed in ccRCC tissues, which could serve as a diagnostic biomarker for ccRCC. DGCR5 isoform-1 could promote ccRCC cell proliferation, migration, and invasion, which is, at least partially, through sponging the tumor-suppressive miR-211-5p to regulate the expression of EMT key component Snail protein as well as its downstream targets, E-cadherin and CLDN7. Thus, DGCR5 isoform-1 can be a diagnostic biomarker and a novel therapeutic target in ccRCC.

## Data Availability Statement

The original contributions presented in the study are included in the article/Supplementary Material, further inquiries can be directed to the corresponding author.

## Ethics Statement

The studies involving human participants were reviewed and approved by the Ethics Committee of Qilu Hospital of Shandong University. The patients/participants provided their written informed consent to participate in this study.

## Author Contributions

XZ and Z-HX designed the experiment. G-XZ, JW, DL, and Y-JF performed the experiments, collected, and analyzed the data. G-XZ performed the bioinformatic analysis under the guidance of B-QL and XZ. JW and XZ contributed to the interpretation of the results. XZ, JW, and G-XZ contributed to the writing and revision of the manuscript. All authors read and approved the final manuscript.

## Conflict of Interest

The authors declare that the research was conducted in the absence of any commercial or financial relationships that could be construed as a potential conflict of interest.

## Footnotes

1 http://bioinformatics.psb.ugent.be/webtools/Venn/

2 https://www.oncomine.org/

## Supplementary Material

Supplementary Figure 1 Primer sets illustration of DGCR5, full-length sequence of the main isoform of DGCR5 and Oncomine data. **(A)** Primer sets targeting different exons of DGCR5. **(B)** The relative DGCR5 expression in RCC cell lines was detected by PCR followed by agarose gel electrophoresis. **(C)** Full-length sequence of the main isoforms of DGCR5. **(D)** The expression of DGCR5 in RCC tissues in Jones and Yusenko cohorts from Oncomine.

Supplementary Figure 2 siRNA-mediated DGCR5 isoform-1 silencing in A704 cells. **(A)** The relative DGCR5 expression in A704 cells transfected with si664, si523, or si1133 targeting DGCR5. qPCR was performed to test the DGCR5 mRNA levels. **(B)** The transfection efficacy of siRNAs. A704 cells transfected with FAM-siRNAs show the green fluorescence in dark field. Independent experiments were performed in triplicate. Error bars represent the mean ± SD; ##*p* < 0.0001.

Supplementary Figure 3 Effects of DGCR5 isoform-1 on RCC cell proliferation and migration. **(A)** DGCR5 mRNA expression detected by qPCR in 786-O and A498 cells. Cells were transfected with si664 targeting DGCR5 or siRNA-NC for 48 h. The mRNA expression of DGCR5 was normalized to GAPDH. Effects of DGCR5 knockdown on cell proliferation and migration, which is detected by CCK8 assay **(B)**, transwell cell migration assay (**C**, magnification ×100), and wound healing assay (**D**, magnification ×100), respectively. Experiments were performed in triplicate in both 786-O and A498 cell lines. Data are shown as mean ± SD; ^∗^*P* < 0.05; #*P* < 0.001. siNC, small interfering RNA negative control; si664, small interfering RNA 664 targeting DGCR5; CCK8, cell counting kit-8; OD, optical density.
