# Supplementary figures and images for: LncRNA DGCR5 Isoform-1 Silencing Suppresses the Malignant Phenotype of Clear Cell Renal Cell Carcinoma via miR-211-5p/Snail Signal Axis
Source: Front Cell Dev Biol. 2021 Jul 12;9:700029. doi: 10.3389/fcell.2021.700029 (PMC8311441; doi:10.3389/fcell.2021.700029)

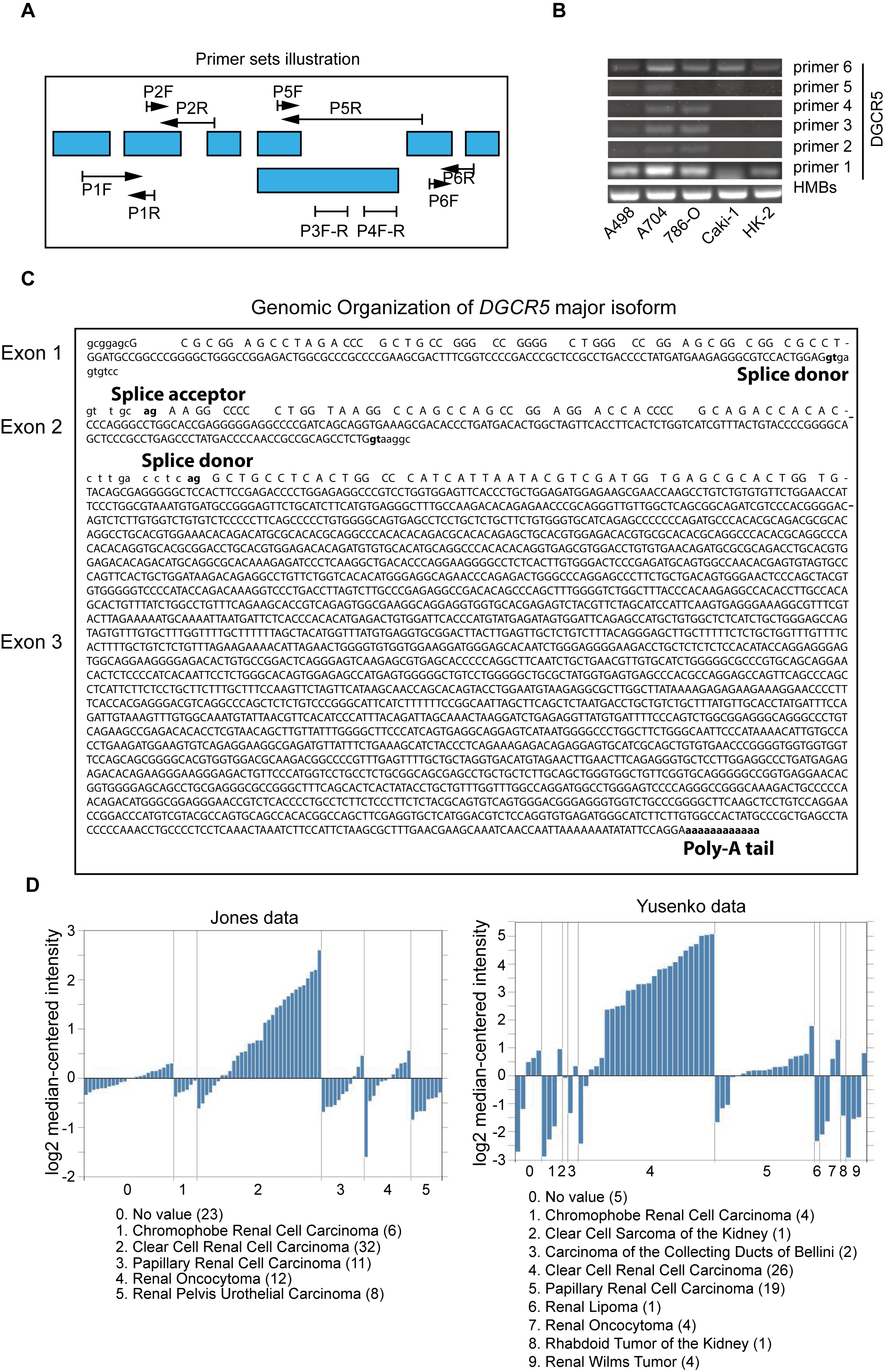

Supplement: Supplementary Figure 1 — Primer sets illustration of DGCR5, full-length sequence of the main isoform of DGCR5 and Oncomine data. (A) Primer sets targeting different exons of DGCR5. (B) The relative DGCR5 expression in RCC cell lines was detected by PCR followed by agarose gel electrophoresis. (C) Full-length sequence of the main isoforms of DGCR5. (D) The expression of DGCR5 in RCC tissues in Jones and Yusenko cohorts from Oncomine. [file Image_1.TIF]

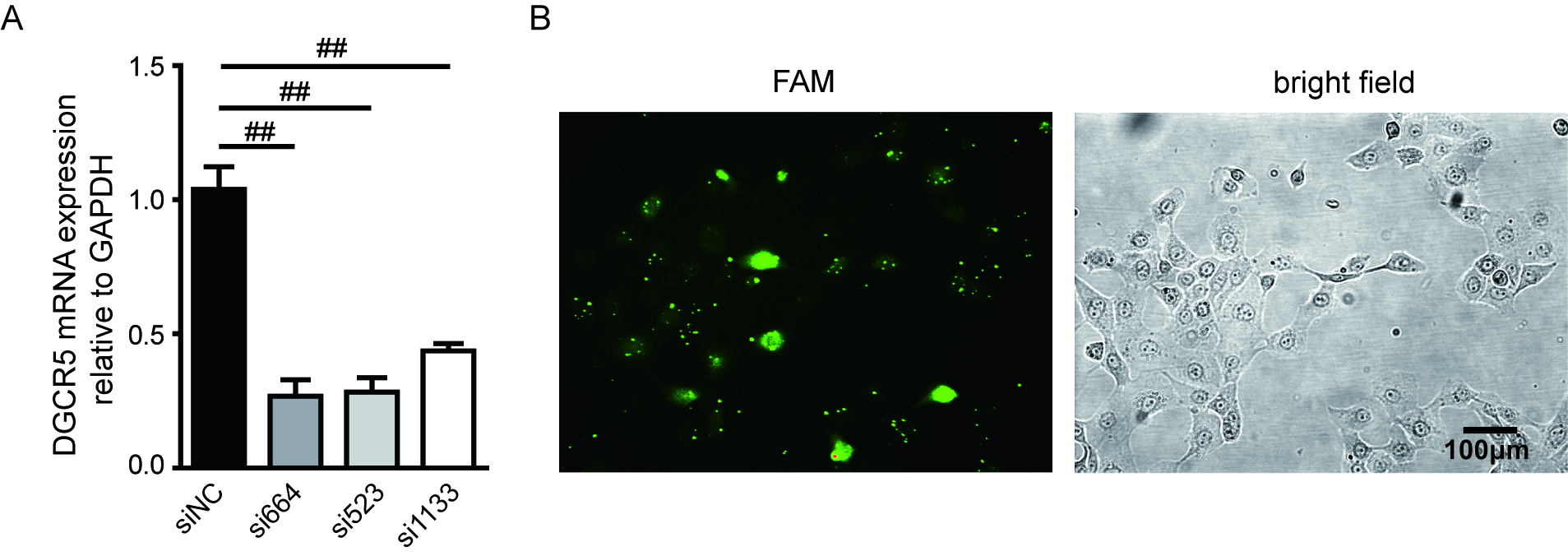

Supplement: Supplementary Figure 2 — siRNA-mediated DGCR5 isoform-1 silencing in A704 cells. (A) The relative DGCR5 expression in A704 cells transfected with si664, si523, or si1133 targeting DGCR5. qPCR was performed to test the DGCR5 mRNA levels. (B) The transfection efficacy of siRNAs. A704 cells transfected with FAM-siRNAs show the green fluorescence in dark field. Independent experiments were performed in triplicate. Error bars represent the mean ± SD; ##p < 0.0001. [file Image_2.TIF]

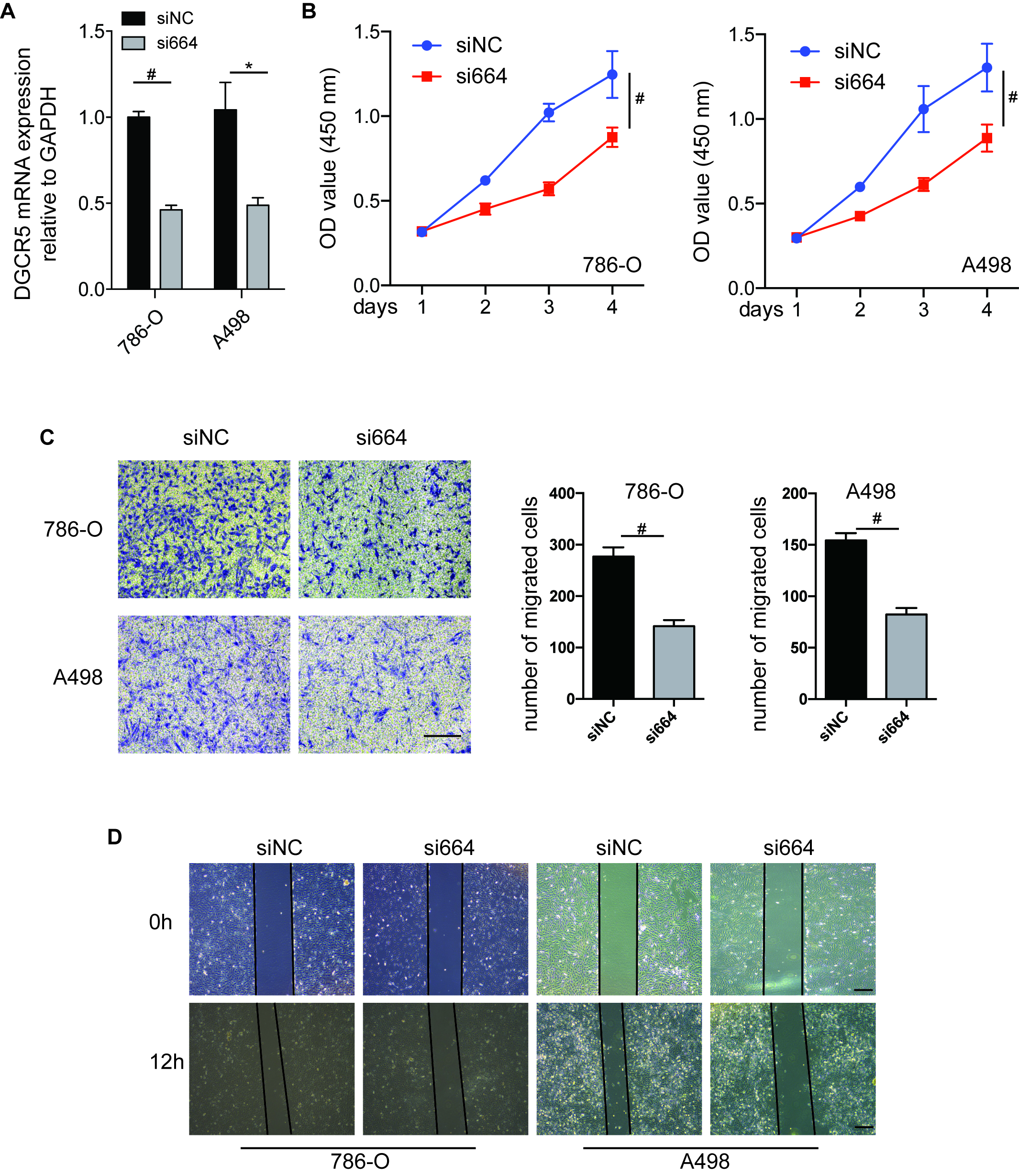

Supplement: Supplementary Figure 3 — Effects of DGCR5 isoform-1 on RCC cell proliferation and migration. (A) DGCR5 mRNA expression detected by qPCR in 786-O and A498 cells. Cells were transfected with si664 targeting DGCR5 or siRNA-NC for 48 h. The mRNA expression of DGCR5 was normalized to GAPDH. Effects of DGCR5 knockdown on cell proliferation and migration, which is detected by CCK8 assay (B), transwell cell migration assay (C, magnification ×100), and wound healing assay (D, magnification ×100), respectively. Experiments were performed in triplicate in both 786-O and A498 cell lines. Data are shown as mean ± SD; ∗P < 0.05; #P < 0.001. siNC, small interfering RNA negative control; si664, small interfering RNA 664 targeting DGCR5; CCK8, cell counting kit-8; OD, optical density. [file Image_3.TIF]
