# Supplementary material for: LncRNA DGCR5 Isoform-1 Silencing Suppresses the Malignant Phenotype of Clear Cell Renal Cell Carcinoma via miR-211-5p/Snail Signal Axis
Source: Front Cell Dev Biol. 2021 Jul 12;9:700029. doi: 10.3389/fcell.2021.700029 (PMC8311441; doi:10.3389/fcell.2021.700029)
Supplement: Supplementary file 4 [file Data_Sheet_1.docx]

| **Table S1** Sequences of primers used in this study | | | |  |
| --- | --- | --- | --- | --- |
| DqE1 | Forward | | CGCCCCGAAGCGACTTT |  |
|  | Reverse | | CTCCAGTGGACGCCCTCTTC |  |
| DqE2 | Forward | | CCAGAGTCCCATGGTTCGAC |  |
|  | Reverse | | ACCAGGGGGCCTTCTTTTTC |  |
| DqE3 | Forward | | GGAACCATTCCCTGGCGTAA |  |
|  | Reverse | | GGGTTCTCTGTGTCTTGGCA |  |
| DqE4 | Forward | | GTTCAGCCCAGCCTCATTCT |  |
|  | Reverse | | AGCCAAGCGCCTCTTACATT |  |
| DqE5 | Forward | | TTCTGGAACCATTCCCTGGC |  |
|  | Reverse | | GTTACCCCTCTGGCCATTCC |  |
| DqE6 | Forward | | GGAATGGCCAGAGGGGTAAC |  |
|  | Reverse | | AGTTGGTGCCCTGTGCTAAT |  |
| β-actin | Forward | | CATGTACGTTGCTATCCAGGC |  |
|  | Reverse | | CTCCTTAATGTCACGCACGAT |  |
| U1 | Forward | | GGGAGATACCATGATCACGAAGGT |  |
|  | Reverse | | CCACAAATTATGCAGTCGAGTTTCCC |  |
| GAPDH | Forward | | GCACCGTCAAGGCTGAGAAC |  |
|  | Reverse | | TGGTGAAGACGCCAGTGGA |  |
| miR-211-5p | Forward | | TCGGCAGGTCCCTTTGTCATCC |  |
|  | Reverse | | TGCAGGTCAACTGGTGTCGT |  |
| U6 | Forward | | CTCGCTTCGGCAGCACA |  |
|  | Reverse | | AACGCTTCACGAATTTGCGT |  |
| DspanE1  DspanE3 | Forward | | CGCCCCGAAGCGACTTTC |  |
|  | Reverse | | GGGTTCTCTGTGTCTTGGCA |  |
| DspanE1  DspanE6 | Forward | | CGCCCCGAAGCGACTTTC |  |
|  | Reverse | | CTGGTGCTCTGAAGCCATGC |  |
| HMBs | Forward | | AGCTTGCTCGCATACAGACG |  |
|  | Reverse | | AGCTCCTTGGTAAACAGGCTT |  |
| E-Cadherin | Forward | | GAACGCATTGCCACATACAC |  |
|  | Reverse | | AGCACCTTCCATGACAGACC |  |
| CLDN7 | Forward | | GGAGATCCCAGGTCACACAT |  |
|  | Reverse | | CAGGGTCTGCCCTAGTCATC |  |
| **Table S2** Sequences of small interfering RNAs (siRNAs) and miRNA mimics or inhibitors used in this study | | | |  |
| DGCR5 si664 | | GAACCAUUCCCUGGCGUAATT | |  |
| DGCR5 si523 | | GCCCAUCAUUAAUACGUCGTT | |  |
| DGCR5 si1133 | | GCACAAAGAGAUCCCUCAATT | |  |
| NC/FAM siRNA | | UUCUCCGAACGUGUCACGUTT | |  |
| miR-211-5p mimics | | UUCCCUUUGUCAUCCUUCGC | |  |
| miR-211-5p inhibitors | | GCGAAGGAUGACAAAGGGAA | |  |

| **Table S3** MicroRNAs predicted by miRcode |
| --- |
| miR-503 miR-551a miR-130ac miR-301ab miR-301b miR-454 miR-721 miR-4295 miR-3666 |
| miR-132 miR-212 miR-7 miR-7ab miR-9 miR-9ab miR-135ab miR-135a miR-138 miR-138ab |
| miR-141 miR-200a miR-142 miR-143 miR-1721 miR-4770 miR-144 miR-145 miR-146ac miR-146b |
| miR-148ab miR-152 miR-150 miR-5127 miR-15abc miR-16 miR-16abc miR-195 miR-322 miR-424 |
| miR-497 miR-1907 miR-17 miR-20ab miR-20b miR-93 miR-106ab miR-427 miR-518a miR-519d |
| miR-181abcd miR-4262 miR-182 miR-183 miR-187 miR-18ab miR-4735 miR-190 miR-190ab |
| miR-192 miR-215 miR-193 miR-193b miR-193a miR-199ab miR-19ab miR-1ab miR-206 miR-613 |
| miR-203 miR-204 miR-204b miR-211 miR-208ab miR-21 miR-590 miR-210 miR-214 miR-761 |
| miR-3619 miR-216a miR-216b miR-217 miR-218 miR-218a miR-22 miR-221 miR-222 miR-222ab |
| miR-1928 miR-23abc miR-23b miR-24 miR-24ab miR-26ab miR-1297 miR-4465 miR-27abc miR-27a |
| miR-101 miR-101ab miR-29abcd miR-30abcdef miR-30abe miR-384 miR-31 miR-124 miR-124ab |
| miR-506 miR-338 miR-33a miR-365 miR-33ab miR-33 miR-34ac miR-34bc miR-449abc miR-449c |
| miR-375 miR-383 miR-125a miR-125b miR-351 miR-670 miR-4319 miR-455 miR-128 miR-128ab |
| miR-129 miR-129ab miR-499 |

| **Table S4** MicroRNAs predicted by DIANA tools with score >0.8 |
| --- |
| miR-211 miR-204 miR-4656 miR-5006 miR-4786 miR-4753 miR-933 miR-7156 miR-1587 |
| miR-3149 miR-4755 miR-1827 miR-6509 miR-7152 miR-3618 miR-4677 miR-5691 miR-6805 |
| miR-3190 miR-6883 miR-6868 miR-6504 miR-7157 miR-4725 miR-3064 miR-6769a miR-130b |
| miR-1207 miR-7974 miR-7151 miR-6780b miR-4273 miR-375 miR-2861 miR-30a miR-5008 |
| miR-4699 miR-30d miR-30e miR-4763 miR-5692a miR-6756 miR-3620 miR-1914 miR-1208 |
| miR-550b-2 miR-4276 miR-6804 miR-8079 miR-3187 miR-500b miR-362 miR-5004 miR-3607 |
| miR-769 miR-515 miR-519e miR-4498 miR-297 miR-653 miR-432 miR-1248 miR-1909 miR-3691 |
| miR-7112 miR-6739 miR-4735 miR-4289 miR-144 miR-203a miR-1288 miR-488 miR-2117 |
| miR-3686 miR-1236 miR-6512 miR-5003 miR-2052 miR-934 miR-5092 miR-1283 miR-3942 |
| miR-6744 miR-8063 miR-4418 miR-2110 miR-4698 miR-214 miR-369 miR-5692b miR-5692c |
| miR-3160 miR-556 miR-4635 miR-4632 miR-6829 miR-6807 miR-3591 miR-4687 miR-4729 |
| miR-2278 miR-655 miR-5708 miR-3942 miR-3663 miR-210 miR-3680 miR-454 miR-4727 |
| miR-3163 miR-761 miR-6769b miR-6720 miR-676 miR-1279 miR-190b miR-3619 miR-4267 |
| miR-190a miR-4722 miR-4688 miR-5701 miR-6165 miR-4517 miR-1264 miR-6828 miR-5704 |
| miR-6721 miR-3074 miR-1290 miR-7150 miR-6743 miR-3977 miR-4736 miR-1245b miR-5100 |
| miR-518d miR-550b miR-876 miR-1237 miR-4295 miR-6852 miR-518c miR-6778 miR-149 |
| miR-6832 miR-518f miR-6751 miR-6512 miR-558 miR-654 miR-4486 miR-3922 miR-892b |
| miR-6766 miR-6890 miR-149 miR-5095 miR-518a miR-3121 miR-4677 miR-181b miR-6737 |
| miR-424 miR-4728 miR-181b-2 miR-4316 miR-6785 miR-4279 miR-766 miR-4659b miR-4530 |
| miR-450a miR-5010 miR-548s miR-877 miR-4435 miR-4295 miR-301a miR-6165 miR-4500 |
| miR-526b miR-6506 miR-2113 miR-219b miR-301b hsa-let-7b miR-130a miR-1245a miR-4686 |
| miR-218 miR-30b miR-30c miR-30a miR-30e miR-30d miR-129 miR-4797 miR-130b miR-153 |
| miR-497 miR-4455 miR-518b miR-4446 miR-4271 miR-4659a miR-4790 miR-3124 miR-875 |
| miR-4689 miR-3150a miR-6782 miR-423 miR-4524b miR-6852 miR-4420 miR-922 miR-6848 |
| miR-6510 miR-4794 miR-3161 miR-876 miR-6857 miR-6071 miR-196b miR-588 |

| **Table S5** Common microRNAs after binding sequence analyses |
| --- |
| miR-211 miR-204 miR-144 miR-497 miR-218 miR-214 miR-129 |
